# Pathological AT1R-B2R Protein Aggregation and Preeclampsia

**DOI:** 10.3390/cells10102609

**Published:** 2021-10-01

**Authors:** Ursula Quitterer, Said AbdAlla

**Affiliations:** 1 Molecular Pharmacology, Department of Chemistry and Applied Biosciences, ETH Zurich, Winterthurerstrasse 190, CH-8057 Zürich, Switzerland; said.abdalla@pharma.ethz.ch; 2 Department of Medicine, Institute of Pharmacology and Toxicology, University of Zurich, Winterthurerstrasse 190, CH-8057 Zürich, Switzerland

**Keywords:** preeclampsia, angiotensin II, AGTR1 (angiotensin II receptor type 1), bradykinin, BDKRB2 (bradykinin receptor B2), AT1R-B2R heteromer (protein complex formed of AT1R-B2R), G-protein-coupled receptor, protein aggregation, ARRB (beta-arrestin)

## Abstract

Preeclampsia is one of the most frequent and severe complications of pregnancy. Symptoms of preeclampsia usually occur after 20 weeks of pregnancy and include hypertension and kidney dysfunction with proteinuria. Up to now, delivery of the infant has been the most effective and life-saving treatment to alleviate symptoms of preeclampsia because a causative treatment does not exist, which could prolong a pregnancy complicated with preeclampsia. Preeclampsia is a complex medical condition, which is attributed to a variety of different risk factors and causes. Risk factors account for insufficient placentation and impaired vasculogenesis and finally culminate in this life-threatening condition of pregnancy. Despite progress, many pathomechanisms and causes of preeclampsia are still incompletely understood. In recent years, it was found that excessive protein complex formation between G-protein-coupled receptors is a common sign of preeclampsia. Specifically, the aberrant heteromerization of two vasoactive G-protein-coupled receptors (GPCRs), the angiotensin II AT1 receptor and the bradykinin B2 receptor, is a causative factor of preeclampsia symptoms. Based on this knowledge, inhibition of abnormal GPCR protein complex formation is an experimental treatment approach of preeclampsia. This review summarizes the impact of pathological GPCR protein aggregation on symptoms of preeclampsia and delineates potential new therapeutic targets.

## 1. Introduction

Preeclampsia is a life-threatening complication of pregnancy, which affects about 2–8% of pregnancies world-wide [1,2]. Risk factors of preeclampsia cause insufficient placentation with defective blood vessels and impaired oxygen delivery to the unborn. As a consequence of defective placentation and impaired vasculogenesis, maternal symptoms of preeclampsia evolve, usually after 20 weeks of pregnancy. Monitoring of blood pressure during pregnancy is an important measure of diagnosis because high blood pressure which exceeds 140/90 mm Hg is a common symptom of preeclampsia [3]. Currently, the only treatment of preeclampsia is (premature) delivery of the infant [4].

However, preterm birth is associated with a high risk to the infant and can cause severe complications, which include respiratory dysfunction due to immature lung development and other health problems [5]. Search for pathomechanisms is necessary to develop a mechanistic basis of preeclampsia treatment, which could minimize life-threatening risks and prolong safe pregnancy. While acute preeclampsia symptoms subside after delivery, a pregnancy complicated with preeclampsia is associated with an increased risk of cardiovascular disease, hypertension and renal dysfunction later in life, for the mother and the infant [6,7]. Therefore, treatment approaches are needed, which leverage not only acute preeclampsia symptoms but also minimize late preeclampsia complications of the cardiovascular and renal systems.

Research efforts worldwide identified several factors associated with preeclampsia. An increase in circulating levels of sFlt1 (soluble FMS-like tyrosine kinase 1) and downregulation of PIGF (placental growth factor) levels are diagnostic markers, which assist in the early diagnosis of preeclampsia [8,9]. The involvement of sFlt1 in symptoms of preeclampsia is documented [10] whereas deficiency of PIGF is redundant and does not cause preeclampsia symptoms [11]. Administration of sFlt1 to pregnant rats causes major symptoms of preeclampsia such as endothelial dysfunction, hypertension and proteinuria [10]. However, the primary trigger of abnormal placentation and sFlt1 up-regulation in preeclampsia still remains to be found.

The search for pathomechanisms of preeclampsia elucidated that pathological protein aggregation of vasoactive G-protein-coupled receptors (GPCRs) characterizes pregnancies complicated with preeclampsia [12,13,14]. Aberrant GPCR protein aggregation affects major target organs of preeclampsia, i.e., the placenta, the vasculature, the kidney and circulating blood cells [12,13,14]. Specifically, a protein complex formed by GPCR heteromerization was documented to be a sufficient cause of preeclampsia [14]. This protein complex consists of two G-protein-coupled receptors, which are known to regulate vascular tone, i.e., the angiotensin II AT1 receptor, AGTR1, and the bradykinin B2 receptor, BDKRB2 [15,16]. Targeting this protein complex formation has been shown to be effective in treating symptoms of experimental preeclampsia [14]. Notably, the protein beta-arrestin1 could support the down-regulation of pathological GPCR aggregates by a beta-arrestin-mediated mechanism [14].

Based on these findings, ongoing research efforts aim to develop a treatment of acute preeclampsia symptoms and/or late cardiovascular preeclampsia complications by inhibition of disease-causing GPCR protein aggregation. This review gives an overview of approaches to target symptoms of preeclampsia by interference with pathological GPCR protein aggregation.

## 2. Preeclampsia: Risk Factors, Symptoms and Diagnosis

### 2.1. Risk Factors of Preeclampsia

There are several established risk factors, which are associated with a significantly increased risk of preeclampsia [6,17,18,19,20,21,22]. Frequent risk factors of preeclampsia, which predispose to a high preeclampsia risk, include major cardiovascular and metabolic diseases such as chronic hypertension, chronic kidney disease, hypertensive disease during a previous pregnancy, type 1 and type 2 diabetes, and obesity with a maternal body mass index of >30 kg/m^2^ [17,18,19]. A high preeclampsia risk is also associated with autoimmune diseases, which cause damage to blood vessels, such as systemic lupus erythematosus, or hypercoagulation such as the antiphospholipid syndrome [17,18,19]. Other risk factors that predispose to moderate risk are advanced age (older than 40 years), multiple pregnancies with twins, triplets or other multiples, the first pregnancy, a long interval between two pregnancies of more than ten years, a new paternity with a long interval between two pregnancies, and a family history of preeclampsia [17,18,19]. In addition, ethnicity can affect the risk of preeclampsia because black women have a higher risk compared to other races [20]. Finally, pregnancies that arise from in vitro fertilization with oocyte donation are also associated with an increased risk of preeclampsia [21,22]. Taken together, the preeclampsia risk is increased in women with major cardiovascular and metabolic diseases and/or autoimmune diseases, which predispose them to hypercoagulation and damage to blood vessels by autoimmune reaction.

### 2.2. Major Symptoms of Preeclampsia

Preeclampsia is a hypertensive disorder of pregnancy. Consequently, one of the most important symptoms of preeclampsia is a rise in blood pressure above 140/90 mm Hg, usually after 20 weeks of pregnancy, in women with previously normal blood pressure [18,23]. In addition, hypertensive disorders such as chronic hypertension that were present before pregnancy or before the 20th week of pregnancy often advance to preeclampsia. This increase in blood pressure can be associated with severe headache, and visual disturbances such as a change in vision, blurred vision or loss of vision [23]. Additional warning signs are epigastric pain, nausea and vomiting [23]. These symptoms are directly linked to high blood pressure and pathophysiological mechanisms of preeclampsia. Headache can be attributed to vasospasm of cerebral arteries and/or cerebral edema [24,25]. Visual disturbances are a consequence of impaired blood flow and vasospasm of retinal arteries causing ischemic injury [26]. Epigastric pain, nausea and vomiting are a consequence of hepatic dysfunction as a result of obstructed blood flow to hepatic sinusoids due to vascular constriction and fibrin-like vascular deposits [27,28].

However, hypertension is not the sole symptom of preeclampsia. Concomitantly with hypertension, preeclampsia causes damage to the microvasculature of other organs, such as the kidney [29,30,31]. Vice versa, kidney disease is causally linked to preeclampsia because preexisting kidney disease is a strong risk factor of preeclampsia [30,31]. Preeclampsia-associated renal dysfunction accounts for proteinuria with an increased albumin-creatinine ratio and/or other markers of acute kidney injury [30,31,32]. Together with hypertension, proteinuria is one of the most frequent symptoms of preeclampsia [33]. Notably, the severity of proteinuria is an indicator of adverse outcome of pregnancies complicated with preeclampsia [33]. As a consequence of kidney dysfunction with decreased urine output, edema with shortness of breath can develop [32].

Additional symptoms of severe preeclampsia are liver dysfunction together with abdominal pain, nausea and vomiting [34]. Another frequent symptom of preeclampsia relates to dysfunctional coagulation, which can be a consequence of thrombotic microangiopathy [35,36], Ensuing thrombocytopenia and hemolysis are characteristic features, which are frequently associated with severe preeclampsia [35,36]. In this context, a serious condition, which coexists with preeclampsia in 70–80% of cases, is the HELLP syndrome. HELPP encompasses hemolysis, elevated liver enzymes and low platelet count [37]. Finally, preeclampsia also affects the unborn. As a consequence of insufficient oxygen delivery, fetal growth restriction is frequently associated with preeclampsia [38,39].

Taken together, preeclampsia is characterized by a rise in blood pressure. Associated symptoms are causally linked to the blood pressure rise and the underlying pathophysiology, which triggers a dysfunction of microvascular beads, specifically the kidney, the brain and the liver [29].

### 2.3. Diagnosis of Preeclampsia

To facilitate early diagnosis of preeclampsia, blood pressure monitoring is a well-established measure of prenatal care [3,40,41]. A rise in blood pressure above 140/90 mm Hg usually after 20 weeks of pregnancy in women with previously normal blood pressure is an important diagnostic criterion of preeclampsia. The rise in blood pressure needs to be confirmed twice, at least 4 h apart [3,40,41].

Hypertension is not the sole diagnostic criterion of preeclampsia [42]. There are other hypertensive disorders of pregnancy that need to be differentiated from preeclampsia, e.g., gestational hypertension or chronic hypertension. Gestational hypertension is hypertension, which occurs for the first time during the second half of pregnancy without proteinuria and organ damage. Preeclampsia is also differentiated from chronic hypertension, which is elevated blood pressure greater than 140/90 mm Hg that was present before pregnancy or before the 20th week of pregnancy. Frequently, these hypertensive disorders of pregnancy advance to preeclampsia [18,42].

In preeclampsia, hypertension is associated with other signs of end organ damage. Thus, the diagnosis of preeclampsia with new-onset hypertension is usually accompanied by one or more additional symptoms of organ dysfunction [18,41]. Kidney dysfunction with proteinuria is such a characteristic but not mandatory feature of preeclampsia [18,41]. Therefore, proteinuria with ≥300 mg in a 24-h urine or a spot urine protein-to-creatinine ratio of ≥30 mg/mmol is often used as a major accompanying diagnostic criterion of preeclampsia [41]. Other diagnostic features of organ dysfunction are acute kidney injury with creatinine of ≥90 micromol/L, or symptoms of liver dysfunction with elevated transaminases with or without abdominal pain. Neurological and hematological complications can also reflect organ dysfunction in preeclampsia. The impaired utero-placental blood flow is diagnosed by uterine artery Doppler waveform analysis [43]. Utero-placental dysfunction is a frequent cause of fetal growth restriction or stillbirth [41,43].

Apart from symptom-based diagnosis, the measurement of circulating angiogenic biomarkers of preeclampsia further facilitates the diagnosis. Of specific value is the determination of circulating levels of sFlt1 and PIGF [8,9,44,45]. In preeclampsia, circulating levels of sFlt1 tend to be increased while circulating PIGF levels are reduced [8,9]. The measurement of PIGF can be used for risk stratification and substantially reduces the time to diagnosis of preeclampsia [46,47,48,49]. In some countries, the ratio of sFlt1/PIGF is recommended as diagnostic criterion to differentiate preeclampsia cases from non-preeclamptic pregnancies [50]. Measurement of the sFlt1/PIGF ratio significantly improves clinical precision but the test has a lower power to differentiate between severe or early onset forms of the condition [44,51]. Likewise, low PIGF should not be considered as a sufficient criterion to perform immediate delivery [52]. Taken together, angiogenic biomarker testing combined with risk stratification substantially reduces the time to diagnosis so that suspected preeclampsia can be considered much earlier and even before proteinuria develops [49].

### 2.4. Prevention Therapy of Preeclampsia with Aspirin

Up to now, there has been no treatment for preeclampsia. The only measure to relieve preeclampsia symptoms is the premature delivery of the infant. Therefore, many guidelines world-wide focus on prevention to delay early onset preeclampsia in high-risk pregnancies. In this context, low-dose aspirin is the most widely recommended prevention therapy of preeclampsia [53,54,55,56]. The recommended therapy for pregnancies with high preeclampsia risk consists of the daily intake of aspirin at a dose of ≥100 mg/d starting from week 11 to 14 of gestation [41,53,54,55,56]. In high-risk pregnancies, aspirin therapy can significantly reduce preterm preeclampsia whereas term preeclampsia is not affected [56]. Despite these recommendations, there is still uncertainty regarding the recommended aspirin dose and the identification of those pregnancies who will profit most from the prevention therapy [53].

## 3. Pathomechanisms of Preeclampsia Leading to Hypertension

### 3.1. The Interrelationship between Impaired Arterial Function, Dysfunctional Placentation, and Hypertension

To identify potential targets for preeclampsia treatment, research activities focus on pathomechanisms, which account for major preeclampsia symptoms. Hypertension after week 20 of pregnancy is one of the major symptoms of preeclampsia. The current concept considers defective placentation with ensuing insufficient uteroplacental perfusion as an initiating event of preeclampsia and preeclampsia hypertension [4,57,58,59]. Dysfunctional placentation is thought to develop as a result of insufficient cytotrophoblast invasion of spiral arterioles [59]. The reduced uteroplacental perfusion accounts for placental ischemia [57,58]. Insufficient oxygen delivery to the placenta with hypoxia and cellular ischemia leads to a widespread dysfunction of the maternal vascular endothelium with increased formation of vasoconstrictors, endothelin and thromboxane, and hypersensitivity to angiotensin II AT1 receptor stimulation [57,58,59]. Endothelial dysfunction is accompanied by a decreased formation of vasodilators, NO and prostacyclin [57,58,59]. Systemic endothelial dysfunction and hypoxia also trigger a disbalance of pro-angiogenic and anti-angiogenic factors with predominance of the anti-angiogenic factor sFlt1 [10,60]. The sFlt1 is the soluble receptor for the vascular endothelial growth factor (soluble VEGFR-1), which antagonizes pro-angiogenic effects of VEGF [10]. The net result is exaggerated vasoconstriction and hypertension. Due to insufficient placentation, the high blood pressure becomes detrimental not only to the mother but also does not improve the oxygen delivery to the developing fetus.

The unresolved question remains: What triggers placental dysfunction that ultimately causes preeclampsia hypertension? Emerging evidence suggests that impaired arterial function could in fact precede placental dysfunction [61]. Consequently, placental impairment would be secondary to cardiovascular dysfunction during the pathogenesis of preeclampsia [61,62]. In agreement with this concept, major risk factors of preeclampsia such as cardiovascular and metabolic diseases, cause damage to the vascular bed. Thus, it is the interplay between primary cardiovascular dysfunction and ensuing placental dysfunction, which finally culminates in insufficient uteroplacental perfusion and placental ischemia [62]. The consequence is a widespread damage of the maternal endothelium and endothelial dysfunction. Endothelial dysfunction is a well-known cause of hypertension and end-organ damage, i.e., the typical symptoms of preeclampsia.

### 3.2. The Angiotensin II AT1 Receptor Hypersensitivity of Preeclampsia

Pathologic alterations of the renin angiotensin aldosterone system (RAAS) are important contributors to preeclampsia. Under physiological conditions, the renin angiotensin aldosterone system exerts an indispensable role in the regulation of vascular tone, blood pressure, kidney function, and salt and water homeostasis [63,64,65]. By cleavage of angiotensinogen, the protease renin liberates angiotensin I, which is further processed by the angiotensin-converting enzyme (ACE) into the octapeptide angiotensin II. Angiotensin II is a major vasoconstrictor by activation of the AT1 receptor, AGTR1 [15,63,64]. In addition, the AT1 receptor regulates salt and water homeostasis, either directly or indirectly by stimulation of the release of the mineral corticoid aldosterone [63,64]. Apart from essential physiological functions, overactivation of RAAS has fundamental pathological consequences and contributes to major cardiovascular and metabolic diseases such as hypertension, atherosclerosis, kidney dysfunction, heart failure, diabetes mellitus, and neurodegeneration [64,65,66]. In agreement with these pathological roles, inhibition of AT1 receptor activation by an ACE inhibitor or an AT1 receptor antagonist are well-documented, evidence-based treatment approaches of cardiovascular diseases with or without concomitant metabolic diseases [63,64,65].

Because cardiovascular and metabolic diseases are also major risk factors of preeclampsia, it is not surprising that dysfunctional RAAS and exaggerated AT1 receptor activation exert major pathological roles in preeclampsia [13,66]. Notably, an increased sensitivity of angiotensin II AT1 receptor-stimulated responses is a long-known and well-established feature of pregnancies complicated with preeclampsia [12,13,14,67,68,69,70]. The angiotensin II AT1 receptor hypersensitivity is documented on vascular specimens and circulating blood platelets of pregnant women with preeclampsia [12,13,14,67,68,69,70]. Vascular angiotensin II AT1 receptor hyperactivation not only contributes to hypertension but also to renal dysfunction with proteinuria and placental dysfunction due to impaired vasculogenesis [12,14,67,68,69,70].

While circulating levels of angiotensin II are low and apparently “exhausted” in preeclampsia [71], pregnant women with preeclampsia often develop agonistic autoantibodies against the AT1 receptor [72]. Through the activation of hypersensitive AT1 receptors, these agonistic autoantibodies contribute to preeclampsia hypertension and preeclampsia symptoms [14,72,73,74]. Causality between agonistic autoantibodies to the AT1 receptor and preeclampsia symptoms was confirmed by several experimental models [73,74]. During the pathogenesis of preeclampsia, agonistic AT1 receptor autoantibodies are triggered by hypersensitive AT1 receptors and/or high blood pressure [14,74,75]. Taken together, hypersensitive and hyperactive AT1 receptors account for exaggerated vasoconstriction and high blood pressure. In addition, agonistic AT1 receptor autoantibodies act as potent and sustained activators of hypersensitive AT1 receptors during the pathogenesis of preeclampsia [14,74].

## 4. Pathologic GPCR Protein Aggregation and Preeclampsia

### 4.1. Pathologic Protein Complex Formation between the AT1 Receptor and the B2 Receptor Causes Angiotensin II AT1 Receptor Hypersensitivity of Preeclampsia

In view of the causative role of angiotensin II AT1 receptor hypersensitivity in symptoms of preeclampsia, the question arises what causes the angiotensin II hypersensitivity of preeclampsia.

Several studies showed that angiotensin II AT1 receptor hypersensitivity is caused by aberrant protein complex formation between the AT1 receptor with the bradykinin B2 receptor [12,13,14].

The bradykinin B2 receptor is a ubiquitously expressed G-protein-coupled receptor, whose in vivo functions are dispensable [76,77,78,79]. Physiologic effects of the bradykinin B2 receptor include the release of nitric oxide and prostaglandins from vascular endothelial cells and vasodilation [76,77,78,79]. These features of the bradykinin B2 receptor contribute to blood pressure lowering and cardioprotection, preferably under conditions with increased bradykinin, e.g., when the ACE-mediated degradation of bradykinin is suppressed by an ACE inhibitor [79]. However, under pathological conditions with endothelial dysfunction and endothelial nitric oxide synthase (eNOS) uncoupling, e.g., preeclampsia and atherosclerosis, the endothelial bradykinin B2 receptor stimulates the generation of reactive oxygen species and thereby loses its cardioprotective functions [80,81,82]. As a consequence of endothelial dysfunction, bradykinin B2 receptor-mediated relaxation of myometrial vessels is impaired in preeclampsia [83,84]. Concomitant with endothelial bradykinin B2 receptor dysfunction, systemic levels of the bradykinin-generating enzyme kallikrein as measured by urinary kallikrein are reduced in preeclampsia [85,86,87]. Furthermore, dysregulated bradykinin B2 receptor and endothelial nitric oxide synthase systems were also documented at the feto-maternal interface of clinical preeclampsia specimens, which showed increased bradykinin B2 receptor levels in extravillous trophoblasts [88]. Local increases in bradykinin B2 receptor protein levels in preeclampsia could be caused by decreased bradykinin B2 receptor stimulation with ensuing depressed B2 receptor down-regulation as a consequence of low systemic kallikrein and bradykinin levels in preeclampsia [85,86,87]. Taken together, endothelial bradykinin B2 receptor-stimulated vasodilation is impaired, and kallikrein and bradykinin levels are reduced in preeclampsia hypertension.

**Figure 1:**
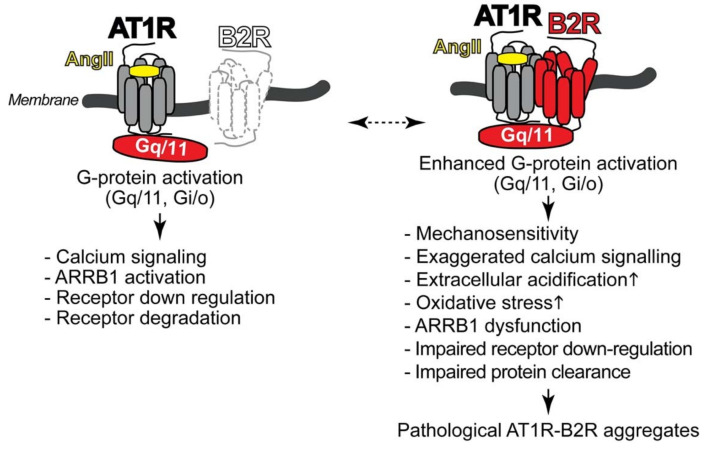
The AT1R-B2R protein complex forms a platform for enhanced G-protein activation. The scheme illustrates how protein complex formation between AT1R (angiotensin II receptor type 1) and B2R (bradykinin B2 receptor) facilitates the interaction with heterotrimeric G-proteins such as Gq/11 (heterotrimeric GTP-binding protein of Gq/11 family) and Gi/o (heterotrimeric GTP-binding protein of Gi/o family). As a consequence of stabilized G-protein interaction, stimulation of the AT1R-B2R protein complex with angiotensin II (AngII) or mechanical stimulation leads to enhanced G-protein activation and signaling, and beta-arrestin1 (ARRB1) dysfunction (**right side**) compared to the monomeric AT1 receptor (**left side**). This Figure was adapted from [90].

In addition, under pathological conditions of preeclampsia, there are increased levels of a heteromeric protein complex between the angiotensin II AT1 receptor and the bradykinin B2 receptor on vascular smooth muscle cells and platelets [12,13,14]. This protein complex formation leads to enhanced G-protein coupling and activation of the AT1 receptor [12,13,14,89]. Notably, the heteromeric AT1R-B2R protein complex between the AT1 receptor and the B2 receptor forms a platform, which facilitates enhanced G-protein activation (Figure 1).

The hypersensitive AT1R-B2R protein complex is distinguished from AT1 receptor monomers by enhanced G-protein activation and a strongly increased mechanosensitivity [14,91]. Consequently, AT1R-B2R can be activated by mechanical forces independently of the agonist angiotensin II. Enhanced G-protein-mediated signal generation by AT1R-B2R accounts for exaggerated calcium signaling (Figure 1). The increased calcium response leads to an increased Na^+^-H^+^-exchanger activation with ensuing increased extracellular acidification [14], which contributes to smooth muscle cell contraction and blood pressure rise [92]. Exaggerated signaling triggered by hypersensitive AT1R-B2R complexes also promotes the increased generation of reactive oxygen species and oxidative stress [12]. Moreover, the enhanced AT1R-B2R-stimulated signaling cascade could be directly involved in beta-arrestin1 (ARRB1) dysfunction of preeclampsia [14]. Dysfunctional ARRB1 not only leads to sustained signaling but also impairs AT1R-B2R down-regulation. Impaired receptor protein down-regulation confers to AT1R-B2R a major feature of pathological protein aggregates, which is dysfunctional protein degradation and impaired protein clearance (Figure 1).

Several lines of evidence support that the B2R-mediated enhancement of angiotensin II-stimulated G-protein activation is mediated by a direct AT1R-B2R protein interaction. First, agonist stimulation of B2R is not essential for the B2R-mediated enhancement of AT1R-stimulated signaling because a mutated B2R (B2R-F297A) with defective agonist binding site also enhances AT1R-stimulated signaling [89]. Second, a mutated B2R (B2R-Y157A) with impaired G-protein activation due to the mutation of Y157A in the conserved DRY motif of B2R is incapable to enhance AT1R-stimulated signaling [89]. Third, shielding of the DRY motif of B2R by site-directed antibodies against the connecting loop between membrane domains III-IV of B2R prevented the angiotensin II-stimulated Gαq/11 (Gq/11 protein alpha subunit) activation and redistribution on maternal vessels of pregnancies complicated with preeclampsia [12]. These preeclamptic vessels were characterized by increased AT1R-B2R protein complexes. Thus, the B2R protein is a sufficient cause of enhanced angiotensin II AT1R-stimulated G-protein activation and signaling.

Some of the above-mentioned features of AT1R-B2R heteromers are reminiscent of obligatory GPCR heteromers such as metabotropic glutamate receptor heteromers or GABAB receptor heteromers [93]. However, there is a major difference between AT1R-B2R and these obligatory GPCR heteromers: In the AT1R-B2R complex, each receptor is fully functional, and acts additively or even synergistically with the other binding partner. Therefore, exaggerated signal generation by AT1R-B2R occurs under pathological conditions with increased AT1R-B2R aggregates such as preeclampsia hypertension. The AT1R can also heteromerize with other GPCRs, such as the angiotensin II receptor type 2, AT2R (AGTR2), or the MAS receptor (MAS1). In contrast to the AT1R signal-sensitizing effect mediated by B2R, the AT2R exerts an antagonistic function and inhibits AT1R-stimulated signaling [94]. Similarly, the interaction of MAS1 with AT1R causes inhibition of AT1R-stimulated functions [95]. On the other hand, the B2R not only enhances signaling stimulated by AT1R via AT1R-B2R heteromerization but also AT2R-mediated effects via AT2R-B2R heteromerization [96]. Depending on the receptor “armamentarium” of a given cell, GPCR heteromerization can thus enhance or depress AT1R-stimulated functions. In preeclampsia hypertension, GPCR heteromerization enhances AT1R-mediated effects.

As a consequence of enhanced signaling, the AT1R-B2R heteromer accounts for increased angiotensin II-stimulated vasoconstriction [12,13,14,89]. Exaggerated vasoconstriction by increased maternal AT1R-B2R heteromers leads to a rise in blood pressure and hypertension at the end of pregnancy in AT1R-B2R-transgenic mice with smooth muscle-specific expression of AT1R-B2R [12,13,14]. Vice versa, mice deficient in B2R (Bdkrb2^−/−^) show depressed angiotensin II AT1R-stimulated vasoconstriction [14,97]. These observations with Bdkrb2^−/−^ mice indicate that under physiological conditions, the B2R may also contribute to angiotensin II AT1R-stimulated vasoconstriction by direct AT1R-B2R interaction. However, the physiological AT1R-B2R heteromerization with endogenously expressed receptor protein levels is different from pathological AT1R-B2R protein complex formation of preeclampsia because AT1R-B2R aggregation in preeclampsia is sustained and practically irreversible. Notably, in preeclampsia, down-regulation of AT1R-B2R protein complexes is impaired, in part due to beta-arrestin1 (ARRB1) dysfunction [12,13,14]. Consequently, the AT1R-B2R protein complex of preeclampsia fulfills criteria of pathological protein aggregates, which accumulate because of dysfunctional protein clearance and/or impaired protein degradation (Figure 1).

Hyperactive AT1R-B2R heteromers trigger enhanced down-stream effects such as the increased generation of vasoconstrictors and endothelin-1 [14,98], which are elevated in preeclampsia [99], and the anti-angiogenic sFlt1, which is a major feature of preeclampsia [8,14]. On placental vessels, the hyperactive AT1-B2 receptor complex contributes to impaired vasculogenesis and placental dysfunction of preeclampsia [12,14]. In the kidney, exaggerated AT1 receptor signaling accounts for kidney dysfunction and proteinuria [14,98]. Preeclampsia symptoms triggered by smooth muscle-specific AT1R-B2R expression also induce hemolysis and low platelet count [14]. Taken together, increased levels of hyperactive heteromeric AT1R-B2R aggregates on target organs of preeclampsia account for angiotensin II hypersensitivity of pregnancies complicated with preeclampsia. Angiotensin II hypersensitivity is a well-established feature of preeclampsia, which has been known for almost fifty years [67].

### 4.2. AT1R-B2R Heteromeric Protein Complexes Trigger Major Symptoms of Preeclampsia

**Figure 2:**
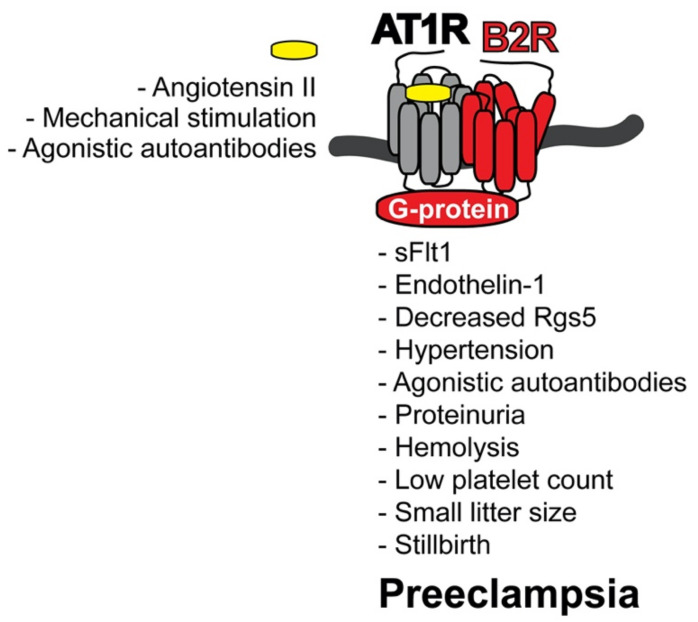
Overview of preeclampsia symptoms triggered by AT1R-B2R. AT1R-B2R triggers major preeclampsia symptoms. Causality between increased AT1R-B2R protein complex formation and preeclampsia symptoms was documented in transgenic mice with smooth muscle-specific AT1R-B2R expression. AT1R-B2R could also cause preeclampsia symptoms in humans because preeclamptic pregnancies are characterized by increased vascular contents of AT1R-B2R aggregation. During the pathogenesis of preeclampsia, AT1R-B2R is activated by angiotensin II, mechanical stimulation and agonistic autoantibodies against AT1R.

Levels of hyperactive AT1R-B2R protein complexes are high in the maternal vasculature of women with pregnancies complicated by preeclampsia [12]. As detailed above, those hyperactive AT1R-B2R protein aggregates on blood vessels account for enhanced angiotensin II-stimulated G-protein-mediated signaling [12,13,14]. Enhanced vascular G-protein-stimulated signaling stimulated by angiotensin II is responsible for vasoconstriction, increased blood pressure and vascular remodeling in animal models and humans [100,101,102]. Increased vascular AT1R-B2R complex levels in transgenic mice with smooth muscle-specific AT1R-B2R expression accounted for a strong blood pressure rise and renal dysfunction with proteinuria at the end of pregnancy [14]. These symptoms are the main features of pregnancies complicated with preeclampsia (Figure 2).

In conjunction with major preeclampsia symptoms (Figure 2), AT1R-B2R protein aggregates triggered a rise in circulating levels of sFlt1 [14]. An increase in sFlt1 is a well-established marker of human preeclampsia [8,9,10,50]. The sFlt1 could be directly induced by AT1R-B2R-stimulated calcium signaling and/or oxidative stress [103,104]. Oxidative stress at the maternal-fetal interface is a typical symptom of preeclampsia [80,105]. Hyperactivation of AT1R and/or AT1R-B2R triggers enhanced calcium signaling, oxidative stress and sFlt1 [14,103,104]. Concomitantly with sFlt1, AT1R-B2R also increased other vasoactive peptides, such as endothelin-1 [14,98]. Increased circulating endothelin-1 levels contribute to preeclampsia symptoms and hypertension [99].

AT1R-B2R also led to reduced vascular Rgs5 (Regulator of G-protein signaling 5) levels [14]. Of note, decreased vascular and placental RGS5 levels are typical features of human pregnancies complicated with preeclampsia [14,106]. The decreased RGS5 level could further enhance the angiotensin II hypersensitivity and preeclampsia hypertension [106].

Together with hypertension, AT1R-B2R triggered AT1R-reactive, agonistic autoantibodies (Figure 2). In pregnant women with preeclampsia, the increased pressure and preeclampsia symptoms are also accompanied by the induction of agonistic autoantibodies against the AT1 receptor [72,73,74,75]. Agonistic AT1 receptor autoantibodies, which are triggered by increased vascular AT1R-B2R heteromers, are most likely induced as a consequence of the preeclampsia-related rise in blood pressure [14].

In addition to hypertension and kidney dysfunction with proteinuria, typical preeclampsia symptoms and end-organ damage were further documented in pregnant AT1R-B2R-transgenic mice by the presence of hemolysis and low platelet count (Figure 2).

In concert with increased levels of circulating sFlt1, enhanced calcium signaling by vascular and placental AT1R-B2R could impair vasculogenesis and cause defective placentation [14]. Likewise, increased vascular AT1R-B2R led to additional preeclampsia symptoms such as intrauterine growth retardation with reduced embryo weights and small litter size [14]. Severe preeclampsia symptoms caused by smooth muscle-specific AT1R-B2R expression also led to stillbirth, as documented by a strongly increased number of dead embryos [14].

Taken together, increased levels of pathological AT1R-B2R protein complexes are a sufficient cause of major preeclampsia symptoms with concomitant rise in antiangiogenic and vasoactive peptides, hypertension, renal dysfunction and end-organ damage in vivo, in transgenic mice with smooth muscle-specific AT1R-B2R expression (Figure 2). Because hyperactive AT1R-B2R heteromers are also increased on maternal and placental vessels of pregnant women with preeclampsia [12,13,14], the AT1R-B2R protein complex could also account for preeclampsia symptoms in human pregnancies complicated with preeclampsia.

### 4.3. Hypersensitive AT1R-B2R Heteromers Are Activated by Mechanical Forces, Which Increase during Pregnancy

**Figure 3:**
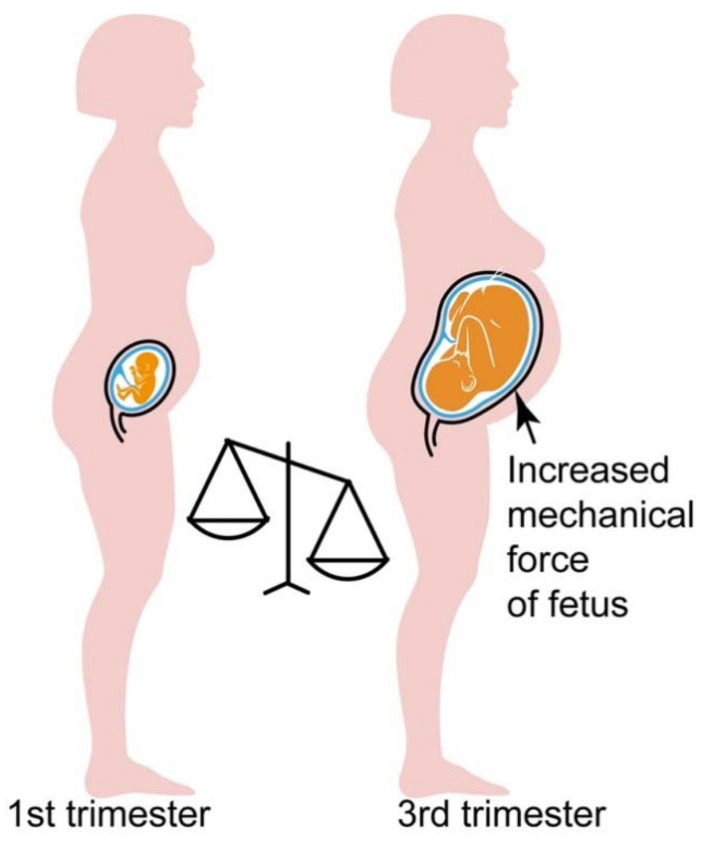
Mechanical forces are high in late-stage pregnancy. Due to the increasing fetal weight, mechanical forces are continuously rising in pregnancy. Consequently, mechanical forces are low in the 1st trimester (**left**), whereas in late-stage pregnancy (3rd trimester), the mechanical force induced by the increased fetal weight is high (**right**).

As a GPCR heteromer, the heteromeric protein complex between the AT1 receptor and the B2 receptor (AT1R-B2R) is activated by the agonist angiotensin II [12,13,14,89,98]. However, circulating angiotensin II levels are depressed in preeclampsia [71]. In addition, plasma levels of the angiotensin II-generating enzyme, ACE, are decreased in pregnancy [107]. Therefore, the question arises, how pathological AT1R-B2R heteromers are activated in pregnancy, when preeclampsia symptoms evolve (Figure 3).

In search for an activator of the AT1R-B2R heteromeric protein complex at the onset of preeclampsia, we found that the ATR-B2R heteromer is also activated without agonist, solely by mechanical forces [14]. Because mechanical activation of AT1R-B2R is agonist-independent, hyperactivation of AT1R-B2R can occur even with low levels of circulating angiotensin II when mechanical forces are high.

During late-stage pregnancy, without and with preeclampsia, mechanical forces are constantly rising due to the increasing weight of the growing fetus [108,109]. Consequently, the AT1R-B2R protein complex is substantially activated by mechanical forces at late-stage pregnancy when mechanical forces are high due to the increased fetal weight (Figure 3).

The third trimester is also the time of onset of preeclampsia symptoms, notably the increased blood pressure. In agreement with involvement of AT1R-B2R in preeclampsia hypertension, a transgenic model with vascular AT1R-B2R expression developed preeclampsia symptoms with elevated blood pressure at late-stage pregnancy [14]. Moreover, twin pregnancies or multifetal pregnancies have an increased preeclampsia risk [109].

By enhancement of AT1R-B2R-mediated signaling, mechano-stimulation of AT1R-B2R by the growing fetus could also actively promote an increase in the preeclampsia-related anti-angiogenic sFlt1 [103,104] and vasoactive endothelin-1 [98,99].

With the increased blood pressure, autoantibodies against the AT1R are triggered, which further stimulate the AT1R-B2R protein complex [14,72,73,74,75]. Thus, the interplay between increasing mechanical forces, agonistic autoantibodies and circulating angiotensin II levels culminate in AT1R-B2R-mediated symptoms of preeclampsia and preeclampsia hypertension at end-stage pregnancy (Figure 3).

### 4.4. Inducers of AT1R-B2R Heteromerization in Preeclampsia

**Figure 4:**
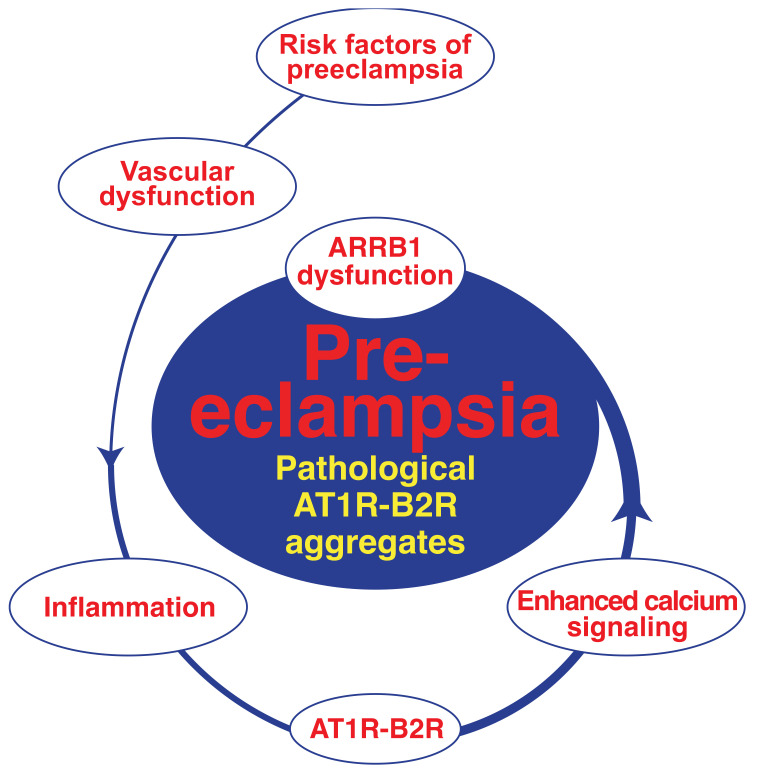
Accumulation of pathological AT1R-B2R aggregates during the pathogenesis of preeclampsia. Pregnancy-induced inflammation triggers AT1R-B2R protein complexes in individuals with major risk factors of preeclampsia and vascular dysfunction. AT1R-B2R stimulates enhanced calcium signaling, aggravates vascular dysfunction and promotes accumulation of pathological AT1R-B2R protein aggregates, in part by ARRB1 dysfunction. At later stages of pregnancy, when mechanical forces are high, pathological AT1R-B2R protein complexes are activated by mechanical forces without angiotensin II, trigger agonistic AT1R autoantibodies and symptoms of preeclampsia.

What triggers AT1R-B2R heteromerization? Anton et al. showed that inflammatory stimuli induced by endotoxin application triggered the formation of the AT1R-B2R protein complex [110]. In agreement with a causal involvement of AT1R-B2R in hypertension, the inflammation-induced AT1R-B2R also led to high blood pressure in the experimental rat model [110]. Inflammation could also trigger the AT1R-B2R heteromer in preeclampsia (Figure 4) because inflammation plays a fundamental role in preeclampsia, and inflammatory markers are high in pregnancies complicated with preeclampsia [111,112].

Concomitant with inflammation-dependent induction of AT1R-B2R, hypersensitive AT1R-B2R aggregates are stabilized during the pathogenesis of preeclampsia because preeclampsia and AT1R-B2R promote the dysfunction of beta-arrestin1 (ARRB1). ARRB1 is known for its involvement in desensitization and down-regulation of AT1R and AT1R-B2R [113,114]. AT1R-B2R could directly stimulate ARRB1 dysfunction by ERK1/2-mediated phosphorylation on serine 412 [14,115]. ERK1/2-stimulated signaling and levels of dysfunctional ARRB1 are increased on vascular cells of the experimental AT1R-B2R-induced preeclampsia model, and on placentas of pregnancies complicated with preeclampsia [14,102]. Consequently, during the pathogenesis of preeclampsia, inflammation-induced AT1R-B2R protein complexes stimulate ARRB1 dysfunction, which in turn promotes pathological AT1R-B2R protein aggregation by protein stabilization (Figure 4).

In addition, the AT1R-B2R-mediated signal enhancement further increases levels of the AT1R-B2R protein complex because the bradykinin B2 receptor gene is a direct target of angiotensin II-stimulated AT1R-mediated signaling [116]. With local B2 receptor induction in cells with AT1 receptor expression and signaling, AT1R-B2R protein complex levels also increase [12,89]. Thus, in frame of the pathogenesis of preeclampsia, AT1R-B2R protein complex formation is initially triggered by inflammation. The thereby enforced AT1R-B2R-mediated signaling triggers more AT1R-B2R by B2R induction and AT1R-B2R protein stabilization as a consequence of AT1R-B2R-mediated ARRB1 impairment. Ensuing accumulation of pathological AT1R-B2R aggregates triggers vascular and placental dysfunction and finally symptoms of preeclampsia (Figure 4).

How is AT1R-B2R protein complex formation related to established risk factors of preeclampsia? The transgenic mouse model of AT1R-B2R-induced preeclampsia develops vascular dysfunction [14]. In this respect, AT1R-B2R-transgenic mice mimic major established risk factors of preeclampsia, which also trigger vascular dysfunction, e.g., hypertension, diabetes, obesity, atherosclerosis, and autoimmune diseases, which cause damage to blood vessels, such as systemic lupus erythematosus, or hyper-coagulation such as the antiphospholipid syndrome [17,18,19]. Vascular dysfunction could impair spiral artery remodeling in early pregnancy [14,117]. Pregnancy-induced inflammation could trigger AT1R-B2R [110], and thereby further aggravate vascular dysfunction. Increased AT1R-B2R stimulates enhances calcium signaling, which promotes the transformation of AT1R-B2R complexes into pathological AT1R-B2R protein aggregates, in part by ARRB1 dysfunction. These pathological AT1R-B2R protein complexes are activated independently of angiotensin II by mechanical forces, when mechanical forces are high at later stages of pregnancy. Thereby, pathological AT1R-B2R complexes promote the preeclampsia-related rise in blood pressure, trigger agonistic AT1R autoantibodies and symptoms of preeclampsia (Figure 4).

## 5. Treatment Approaches of Preeclampsia

### 5.1. Aspirin for Prevention of Preeclampsia

There are very few treatment options for preeclampsia symptoms with documented efficacy. The most effective treatment for acute preeclampsia symptoms is still premature delivery of the baby. Nevertheless, numerous efforts and clinical studies were performed worldwide, which aim to reduce the risk of preeclampsia by pharmacological approaches. Currently, one of the best documented options is the use of low-dose aspirin for the extension of pregnancies at high risk of preeclampsia [53,54,55,56]. The introduction of aspirin was however highly controversial. Early data from 1985 showed the first evidence that low-dose aspirin could retard symptoms of preeclampsia [118]. It took more than 30 years until large placebo-controlled trials could confirm the therapeutic benefit of aspirin for prevention of preeclampsia in high-risk pregnancies with a history of preeclampsia [119]. According to currently available clinical studies, aspirin at a low dose of 80 to 150 mg/d started at 12 weeks of gestation could reduce the risk of preeclampsia in frame of secondary prevention in pregnant women with a history of preeclampsia [53,54,55,56]. Treatment side effects such as abruptio placentae could be a problem in nulliparous women [53,54,55,56]. Ongoing efforts aim to define the optimum aspirin dose, which could be higher than the currently recommended dose [120].

The mechanism of action of aspirin includes the inhibition of thromboxane A2 and platelet aggregation [119]. When given at a low dose, aspirin selectively inhibits platelet-derived thromboxane whereas endothelium-derived prostacyclin is maintained [119]. Notably, preeclampsia is characterized by increased thromboxane A2 and depressed prostacyclin levels [119]. This imbalance of thromboxane A2 versus prostacyclin is present from 13 weeks of gestation on [119]. Therefore, aspirin shows the highest benefit when it starts at about 12–13 weeks of gestation [53,54,55,56,119]. It seems to be of little value when started later, at 16 weeks of gestation [53,54,55,56,119]. Taken together, aspirin is currently one of the best documented pharmacological treatment options for risk reduction in preeclampsia and extension of pregnancy duration in pregnancies at high risk of preeclampsia.

Increased AT1R-B2R protein complex formation also exerts a major role in dysfunctional platelet aggregation of preeclamptic women. Platelets isolated from women with pregnancies complicated by preeclampsia display angiotensin II AT1R hypersensitivity [68], and increased AT1R-B2R heteromerization accounts for angiotensin II AT1R hypersensitivity of platelets [12]. Causality between AT1R-B2R and platelet dysfunction is further documented by the fact that the AT1R-B2R-induced preeclampsia model develops not only preeclampsia symptoms but also low platelet count and hemolysis [14]. Furthermore, the platelet aggregation-enhancing function of angiotensin II AT1R-stimulated signaling is well-documented in vitro and in vivo [121,122,123]. Therefore, preeclampsia prevention with aspirin is also expected to counteract the AT1R-B2R-enhanced platelet dysfunction of preeclampsia.

### 5.2. Targeting of Preeclampsia Symptoms and AT1R-B2R by Beta-Arrestin-Biased Agonism at the AT1 Receptor

**Figure 5:**
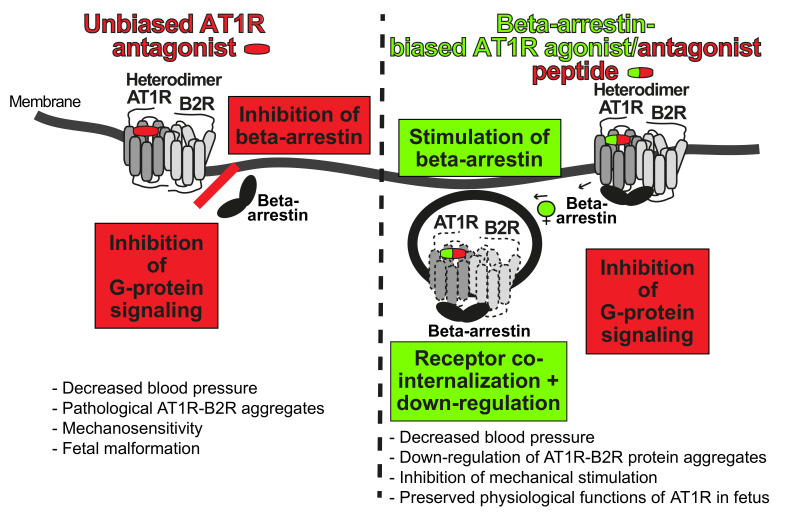
Major differences between unbiased AT1R antagonists and beta-arrestin-biased AT1R agonists/antagonists. An unbiased AT1R antagonist such as losartan inhibits (red color) both, G-protein signaling and beta-arrestin recruitment to the AT1R-B2R complex (**left side**). A beta-arrestin-biased AT1R agonist/antagonist peptide inhibits G-protein signaling (red color) but stimulates beta-arrestin recruitment (green color) to the AT1R-B2R complex. Thereby, the beta-arrestin-biased AT1R agonist/antagonist stimulates AT1R-B2R co-internalization (indicated by arrows) and down-regulation (**right side**). This Figure was adapted from [90].

Based on the causal role of AT1R-B2R in the pathogenesis of preeclampsia, the AT1R-B2R protein complex is an emerging therapeutic target of preeclampsia (Figure 5). Several targeting strategies are conceivable. The most straightforward approach would be the direct inhibition of the angiotensin II-stimulated AT1R-B2R by an AT1R antagonist. However, direct inhibition of the angiotensin II system is not feasible in preeclampsia because the RAAS exerts an indispensable role in fetal development, notably in the second and third trimester [124,125]. Therefore, direct inhibition of angiotensin II by an ACE inhibitor or AT1R antagonist is contraindicated in pregnancy because of fetal side effects such as oligohydramnios [124,125].

A different approach of AT1R-B2R targeting is based on the role of beta-arrestin, ARRB (1), in GPCR and AT1R-B2R down-regulation [14,113]. The concept relies on beta-arrestin-biased agonism [126,127,128,129,130]. Among other effects, beta-arrestin-biased agonists stimulate beta-arrestin-mediated GPCR internalization and down-regulation [126,127,128,129,130]. By stimulating beta-arrestin-mediated GPCR down-regulation, beta-arrestin-biased agonists have a bias for beta-arrestin but do not activate G-protein-stimulated signaling (Figure 5). With these characteristics, a beta-arrestin-biased agonist at the AT1 receptor stimulates AT1R-B2R downregulation and prevents hyperactive angiotensin II AT1R-B2R-stimulated G-protein activation and signaling [14,113]. By AT1R-B2R down-regulation, the beta-arrestin-biased agonist, [Sar^1^,Ile^4^,Ile^8^]-AngII, also blocks stimulation of AT1R-B2R by mechanical forces [14]. For comparison, the non-biased AT1 receptor antagonist, losartan, not only blocks AT1R-B2R-stimulated receptor signaling but also prevents beta-arrestin-dependent receptor down-regulation [14]. Moreover, and in contrast to the biased agonist, the unbiased antagonist losartan does not block signaling of AT1R-B2R aggregates stimulated by mechanical forces [14].

In contrast to unbiased AT1R antagonists, beta-arrestin-biased agonists/antagonists at the AT1 receptor not only promote AT1R-B2R co-internalization and down-regulation in vitro, in cultured cells, but also in vivo [14,113]. The beta-arrestin-biased AT1R agonist, TRV027, prevented preeclampsia-induced hypertension and AT1R-B2R hyperactivation-induced symptoms of preeclampsia in an experimental AT1R-B2R-induced preeclampsia model [14]. The beta-arrestin-biased AT1R agonist, TRV027, also lowered blood pressure in several other experimental models of hypertension [131,132]. Thus, blood pressure-lowering and down-regulation of AT1R-B2R protein aggregates in vivo is feasible by a beta-arrestin-biased AT1R agonist [14,131,132]. In addition, targeting of AT1R-B2R by beta-arrestin-biased agonism not only lowers blood pressure but also prevents symptoms of preeclampsia [14].

Currently available beta-arrestin-biased AT1R agonists are peptides with a short half-life and a low oral bioavailability. The first beta-arrestin-biased AT1R agonist tested for clinical use, TRV027, has a favorable side-effect profile in humans, in a phase-II clinical study [133]. These features could be relevant for the development of biased AT1 receptor agonists for use in preeclampsia because due to the short half-life and low bioavailability, the indispensable fetal AT1 receptor is not fully blocked, and physiological functions of AT1R in the fetus will be preserved.

### 5.3. Inhibition of Exaggerated AT1R-B2R-Stimulated Calcium Signaling of Preeclampsia

Hyperactive AT1R-B2R aggregates stimulate excessive calcium signaling, which contributes to vasoconstriction, aberrant vascular remodeling and dysfunctional placentation during the pathogenesis of preeclampsia [12,13,14]. Inhibition of cellular calcium overload-induced vasoconstriction by an L-type calcium channel antagonist is frequently used to treat preeclampsia-induced hypertension [134]. The comparison of different L-type calcium channel antagonists showed that the long-acting amlodipine not only lowers blood pressure but also decreases AT1R-B2R protein complexes by restoration of beta-arrestin1 function in experimental and human preeclampsia cases [14]. However, due to their powerful antihypertensive activity, L-type calcium channel antagonists are only applicable for preeclampsia cases with high blood pressure increases [134].

An alternative approach could target AT1R (AT1R-B2R), and the L-type calcium channel simultaneously [135]. The experimental strategy uses a bivalent vaccine against the AT1 receptor and the L-type calcium channel, and thereby simultaneously inhibits the AT1 receptor and the Cav 1.2 channels as the major L-type calcium channel in humans [135]. The bivalent vaccine effectively lowered blood pressure and prevented end-organ damage in experimental models of hypertension in mice and rats, without major side effects [135]. A similar approach is conceivable to target the hyperactive AT1R-B2R of individuals at high risk of developing preeclampsia hypertension.

Together, these findings indicate that exaggerated AT1R-B2R protein complex formation of preeclampsia could be targeted by different approaches and chemical entities, e.g., by beta-arrestin-biased AT1R agonists, by inhibition of exaggerated calcium signaling or both. Research efforts are ongoing, which aim to develop specific targeting approaches of pathological AT1R-B2R aggregates and novel AT1R-inhibitory compounds [136]. These strategies could be further exploited to develop a therapy for preeclampsia symptoms, which is safe for the mother and infant.

## 6. Long-Term Complications of Preeclampsia

### 6.1. Preeclampsia Increases the Risk of Cardiovascular and Renal Dysfunction Later in Life

Despite enormous efforts worldwide, delivery is still the most effective therapy for preeclampsia symptoms. (Preterm) delivery usually treats the acute maternal symptoms of preeclampsia. However, pregnancies complicated with preeclampsia are associated with an increased risk of cardiovascular disease and kidney dysfunction later in life [7,137,138]. This association was already recognized early in the 19th century and in the 1960s and 1970s of the 20th century [137]. Meanwhile, a panoply of different studies worldwide document that a woman with a pregnancy complicated with preeclampsia is at increased risk to develop chronic hypertension and renal dysfunction during lifetime [137,138]. With a history of preeclampsia, the risk of cardiovascular disease is increased 2-fold, and the risk of end-stage renal disease is increased up to 5–10-fold [138]. The increased risk of cardiovascular and/or renal disease after preeclampsia is not fully understood. On one hand, pregnancy complications such as preeclampsia, could unmask a preexisting elevated risk of cardiovascular damage in these women [139]. On the other hand, the preeclampsia-induced damage could initiate pathophysiological changes that trigger cardiovascular disease later in life [139].

Preeclampsia does not only affect the cardiovascular health of the mother but also of the infant. Children born after a pregnancy complicated with preeclampsia usually have a low birth-weight. Preterm birth with low birth weight is known to be associated with a high risk of cardiovascular disease later in life, and an increased cardiovascular mortality [6,140]. Apart from prematurity, increasing evidence suggests that preeclampsia has an extra impact on the cardiovascular health of the offspring [140,141]. The increased risk of hypertension and stroke later in life indicates that preeclampsia has a yet unexplored impact on cardiovascular health of the infant, which cannot solely be explained by premature birth alone. Most likely, preeclampsia is associated with substantial cardiac and vascular alterations in the mother and the offspring, all of which are poorly understood.

### 6.2. AT1R-B2R Aggregation Increases the Risk of Renal Dysfunction as a Long-Term Complication of Preeclampsia

Although delivery of the infant relieves acute symptoms of preeclampsia, women with a pregnancy complicated by preeclampsia have an increased risk of developing cardiovascular and renal disease later in life.

Notably, a major long-term complication of preeclampsia is an increased risk of renal failure [138,142,143,144,145,146]. Several recent meta-analyses provide strong evidence that women with a pregnancy complicated with preeclampsia have a strong, up to 5–12-fold increased risk to develop end-stage kidney disease [139,142,143,144,145,146]. Until now, data came from meta-analyses. Those study results suggest that a subset of women with a history of preeclampsia have a highly increased risk of kidney disease [144]. The risk of kidney disease could be associated with the severity and/or subtype of preeclampsia because early onset preeclampsia seems to be associated with a higher risk of renal disease within 5 years after preeclampsia than that of late-onset preeclampsia [144]. The increased risk of renal failure after preeclampsia is at least partially associated with chronic hypertension because preeclampsia superimposed by chronic hypertension confers a substantially increased risk of end-stage renal disease compared to preeclampsia alone [145]. In addition, the risk of end-stage kidney disease is substantially enhanced after more than one pregnancy complicated with preeclampsia [146].

**Figure 6:**
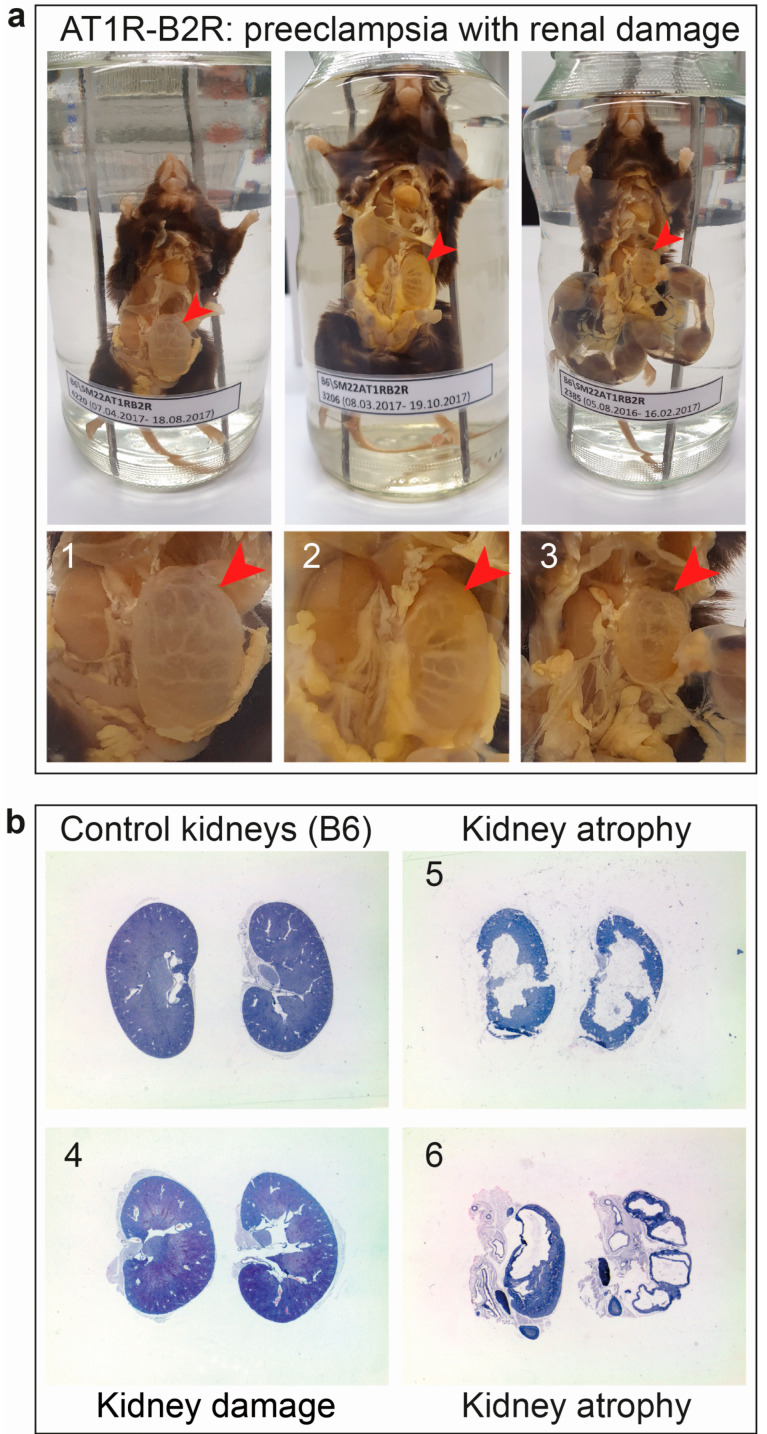
Kidney damage after multiple preeclamptic pregnancies in AT1R-B2R-transgenic mice. (**a**) In situ examination shows preeclampsia symptoms with uterine hemorrhage and abortion (upper panels), and kidney damage (lower panels) of female AT1R-B2R-transgenic mice (no. 1–3; age: 4–7 months); (**b**) Kidney damage and renal atrophy was detected by histological analysis of hematoxylin-eosin-stained kidney specimens of three female AT1R-B2R-transgenic mice after multiple preeclamptic pregnancies (no. 4–6; age 4–7 months). The left upper panel shows control kidney specimens of a non-transgenic, age-matched, female B6 mouse with uncomplicated pregnancies.

The experimental model of AT1R-B2R-induced preeclampsia also shows renal complications in the long term. In situ examination and histological analyses found that 30% of female mice with increased vascular AT1R-B2R heteromerization and several pregnancies complicated with preeclampsia had developed kidney atrophy (Figure 6). These findings complement data from human studies, which show that the risk of end-stage kidney disease rises substantially after several pregnancies complicated with preeclampsia [146]. Therefore, therapeutic approaches to inhibit and down-regulate AT1R-B2R aggregates aim not only to alleviate acute preeclampsia symptoms but also counteract and prevent long-term complications of preeclampsia, and other disorders associated with increased pathological AT1R-B2R aggregation.

### 6.3. Outlook

Increased pathological AT1R-B2R aggregation is a major contributor to angiotensin II hypersensitivity of preeclampsia and a sufficient cause of major preeclampsia symptoms in vivo. Because biopsy specimens of women with pregnancies complicated by preeclampsia are also characterized by an increased AT1R-B2R protein complex formation [12,14], a causative role of AT1R-B2R in human preeclampsia is strongly suggested. Based on the causal involvement in major preeclampsia symptoms, pathological AT1R-B2R protein complexes could be exploited as a pharmacological target to develop a treatment strategy. Such a treatment could delay the onset of acute preeclampsia symptoms in individuals with increased AT1R-B2R aggregation. In addition, inhibition of pathological AT1R-B2R aggregates by pharmacological approaches could also constitute a potential strategy to prevent long-term complications of preeclampsia in the mother and offspring.

## Data Availability

All data presented are contained within the article.
